# Region-Specific Enhancement of c-fos Expression by Combined Treatment With NMDA Receptor Agonists and Antagonists With Antidepressant Potential

**DOI:** 10.1093/ijnp/pyac051

**Published:** 2022-08-17

**Authors:** Andrei-Nicolae Vasilescu, Natascha Pfeiffer, Federica Terraneo, Marco Andrea Riva, Undine E Lang, Dragos Inta, Peter Gass

**Affiliations:** RG Animal Models in Psychiatry, Department of Psychiatry and Psychotherapy, Medical Faculty Mannheim, Central Institute of Mental Health, Mannheim Faculty, Heidelberg University, Mannheim, Germany; RG Animal Models in Psychiatry, Department of Psychiatry and Psychotherapy, Medical Faculty Mannheim, Central Institute of Mental Health, Mannheim Faculty, Heidelberg University, Mannheim, Germany; Department of Pharmacological and Biomolecular Sciences, University of Milan, Milan, Italy; Department of Pharmacological and Biomolecular Sciences, University of Milan, Milan, Italy; Biological Psychiatry Unit, IRCCS Istituto Centro San Giovanni di Dio Fatebenefratelli, Brescia, Italy; Department of Psychiatry (UPK), University of Basel, Basel, Switzerland; RG Animal Models in Psychiatry, Department of Psychiatry and Psychotherapy, Medical Faculty Mannheim, Central Institute of Mental Health, Mannheim Faculty, Heidelberg University, Mannheim, Germany; Department for Community Health, Faculty of Natural Sciences and Medicine, University of Fribourg, Fribourg, Switzerland; RG Animal Models in Psychiatry, Department of Psychiatry and Psychotherapy, Medical Faculty Mannheim, Central Institute of Mental Health, Mannheim Faculty, Heidelberg University, Mannheim, Germany

**Keywords:** NMDA receptors, antidepressant drug, MK-801, Rapastinel, D-Cycloserine

## Abstract

Rapastinel, formerly Glyx-13, is a novel positive allosteric modulator of the N-methyl-D-aspartate-receptor (NMDAR) that counteracts psychotomimetic actions of NMDAR antagonists. We set out to evaluate the effect of rapastinel alone or in combination with the global and GluN2B subunit–specific NMDAR antagonists MK-801 and Ro25-6981, respectively, on neuronal activation in relevant regions using c-fos brain mapping. Whereas rapastinel alone did not trigger significant c-fos expression beyond the prelimbic cortex, it strongly increased the c-fos expression induced by MK-801 in hippocampal, cingulate, and retrosplenial areas. Similar results were obtained when rapastinel was replaced by D-cycloserine. Our results reveal new interactions at network level between NMDAR modulators with possible implications regarding their therapeutic effects.

## Introduction

Glutamatergic agents are increasingly studied as a possible treatment avenue in depression and schizophrenia, and recently esketamine has been approved as an adjunctive therapy in treatment-resistant depression (Salahudeen, Wright and Peterson 2020). However, its use is hampered by side effects, including psychosis (Carboni et al. 2021). Rapastinel is a novel N-methyl-D-aspartate-receptor (NMDAR) modulator that enhances NMDAR function and displays fast antidepressant and antipsychotic actions without the side effects of ketamine (Vasilescu et al., 2017). The mechanisms underlying the effects of these glutamatergic modulators are not entirely understood. Recent results indicate a main role of NMDAR-subunit GluN2B on neuronal populations in mediating their antidepressant effect: cell-type-specific knockdown (KD) of NMDAR-GluN2B on glutamatergic neurons blocks the antidepressant effects of rapastinel, whereas GluN2B KD on GABAergic interneurons blocks the actions of ketamine (Pothula et al., 2021). Moreover, rapastinel directly enhances NMDAR activity independent of the NMDAR glycine site or of D-serine or glycine site antagonists (Donello et al., 2019), suggesting a unique binding profile.

We aimed to evaluate the network effects of rapastinel, focusing on brain regions involved in depression and psychosis. Using c-fos mapping, we determined the effect of rapastinel alone or in combination with the global and GluN2B subunit-specific NMDAR antagonists MK-801 and Ro25-6981, respectively. We also compared the effect of rapastinel on c-fos expression with that of D-Cycloserine, an NMDAR modulator binding at the glycine site (Matsuoka and Aigner 1996).

## MATERIALS AND METHODS

Experiments were performed on 2 groups of adult C57BL6 mice (n = 84) purchased from Charles River, Sulzfeld, Germany as breeding pairs, as previously described (Inta et al., 2009). The first group was treated with either (A) vehicle (0.9% NaCl, 5 mL/kg i.p., n = 7), (B) Ro 25-6981 (10 mg/kg, n = 7), (C) rapastinel (10 mg/kg i.p., n = 10), (D) rapastinel + Ro 25-6981 (10 mg/kg + 10 mg/kg i.p., n = 8), (E) MK-801 (0.5 mg/kg i.p., n = 10), or (F) rapastinel + MK-801 (10 mg/kg + 0.5 mg/kg i.p., n = 10). Animals of the second group were administered either (A) vehicle (0.9% NaCl, 5 mL/kg i.p., n = 7), (B) D-cycloserine (20 mg/kg, n = 7), (C) rapastinel + MK-801 (10 mg/kg + 0.5 mg/kg i.p., n = 10), or (D) D-cycloserine + MK-801 (20 mg/kg + 10 mg/kg i.p., n = 8). Two hours after application, the animals were anesthetized and transcardially perfused using 4% paraformaldehyde (in 0.1 M phosphate buffer, pH 7.4). The brains were removed and postfixed for 24 hours, then cut into 5-µm-thick coronal sections. Substance dosages and time of killing were based on previous research (Donello et al., 2019; Inta et al., 2012). All experiments were approved by the local Committee on Animal Care and Use and in accordance with the local Animal Welfare Act and the European Communities Council Directive 2010/63.

Sections were incubated free-floating with anti-c-fos antibodies (monoclonal; Cell Signaling Technology, diluted 1:100). Staining was visualized using nickel-3,3′-diaminobenzidine (Inta et al. 2009). Cell counting was executed using a light microscope (Leica TCS-NT) at 40× magnification in regions of interest (CA1, CA3, dentate gyrus, cingulate cortex, retrosplenial cortex, nucleus accumbens) and was performed in 3–4 adjacent sections for each animal, bilaterally. The average value across all examined sections was determined. The average density for each treatment group was calculated and statistically analyzed.

Statistical analyses were performed using the statistical program SPSS 23 for Windows. The mean number of c-fos–expressing cells was calculated for each treatment group, and differences between groups were evaluated by use of 1-way ANOVA followed by post hoc Bonferroni testing, with *P *< .05 considered statistically significant.

## RESULTS

**Figure 1.**
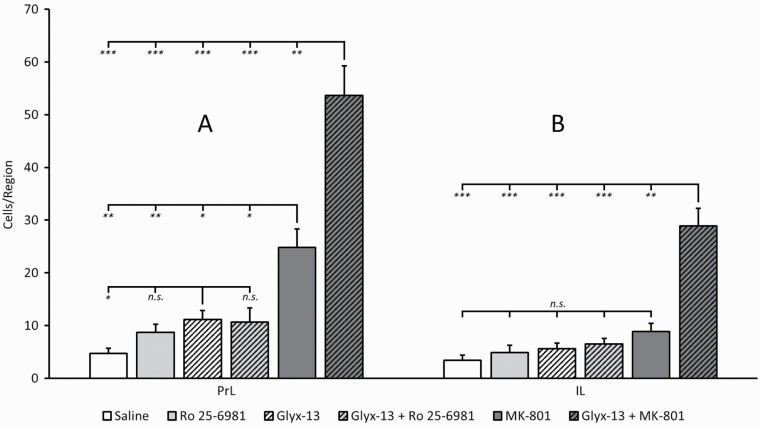
Quantitative analysis of c-fos expression after treatment with saline, Ro 25-6981, rapastinel (Glyx- 13), rapastinel (Glyx-13) + Ro 25-6981, MK-801, or rapastinel (Glyx-13) + MK-801 in the prelimbic cortex (A) (F_5,46_ = 29, *P*<.0001) and infralimbic cortex (B) (F_5,46_ = 26, *P*<.0001). While rapastinel (Glyx-13) alone induced a slight signal increase, mainly in the prelimbic area, this effect was significantly augmented by the combination with MK-801. *P < .05, **P < .01, ***P < .001. n.s., not significant.

**Figure 2.**
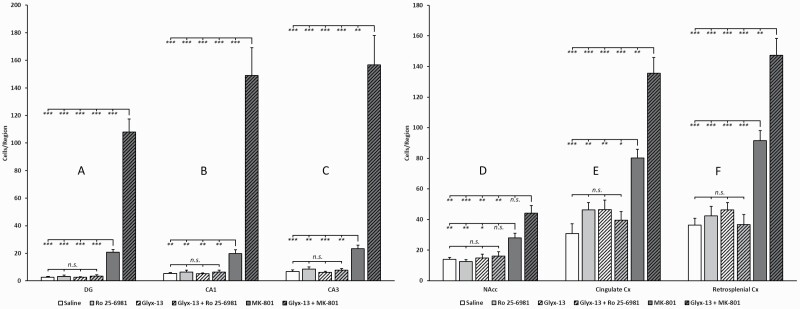
Quantitative analysis of c-fos expression after treatment with saline, Ro 25-6981, rapastinel (Glyx- 13), rapastinel (Glyx-13) + Ro 25-6981, MK-801, or rapastinel (Glyx-13) + MK-801 in the regions of interest dentate gyrus (A) (F_5,46_ = 93, *P*<.0001), CA1 (B) (F_5,46_ = 39, *P*<.0001), CA3 (C) (F_5,46_ = 38, *P *< .0001), nucleus accumbens (D) (F_5,46_ = 13, *P *< .0001), cingulate cortex (E) (F_5,46_ = 30, *P*<.0001), and retrosplenial cortex (F) (F_5,46_ = 37, *P *< .0001). Note the markedly enhanced hippocampal expression induced by the combination rapastinel (Glyx-13) + MK-801 compared with MK-801 and rapastinel, respectively, alone (A–C). Also note the enhanced expression in the cingulate and retrosplenial cortex (E, F) induced by the combination rapastinel (Glyx-13) + MK-801 compared with MK-801 and rapastinel, respectively, alone. In the nucleus accumbens (D), the combination rapastinel (Glyx-13) + MK-801 evoked significantly more c-fos expression than Glyx-13 alone. *P < .05, **P < .01, ***P < .001. n.s., not significant.

**Figure 3.**
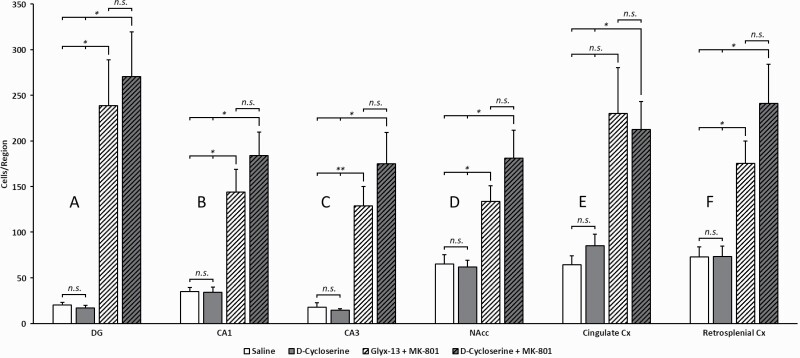
Quantitative analysis of c-fos expression after treatment with saline, D-cycloserine, rapastinel (Glyx-13), rapastinel (Glyx-13) + MK-801, or D-cycloserine + MK-801 in the hippocampal subsectors dentate gyrus (A), CA1 (B), CA3 (C), as well as nucleus accumbens (D), cingulate cortex (E), and retrosplenial cortex (F). Similar to rapastinel, D-cycloserine also leads in combination with MK-801 to a markedly increased c-fos expression in all brain regions studied. *P < .05, **P < .01, ***P < .001. n.s. not significant.

**Figure 4.**
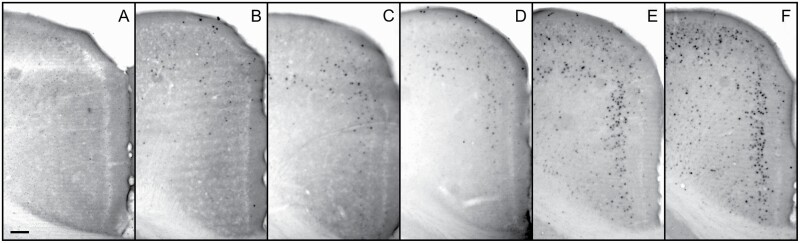
Cropped microscopy images showing c-fos induction in the retrosplenial cortex by treatment with saline (A), Ro 25-6981 (B), rapastinel (Glyx-13) (C), rapastinel (Glyx-13) + Ro 25-6981 (D), MK-801 (E), and rapastinel (Glyx-13) + MK-801 (F). Scale bar = 100 μm.

**Figure 5.**
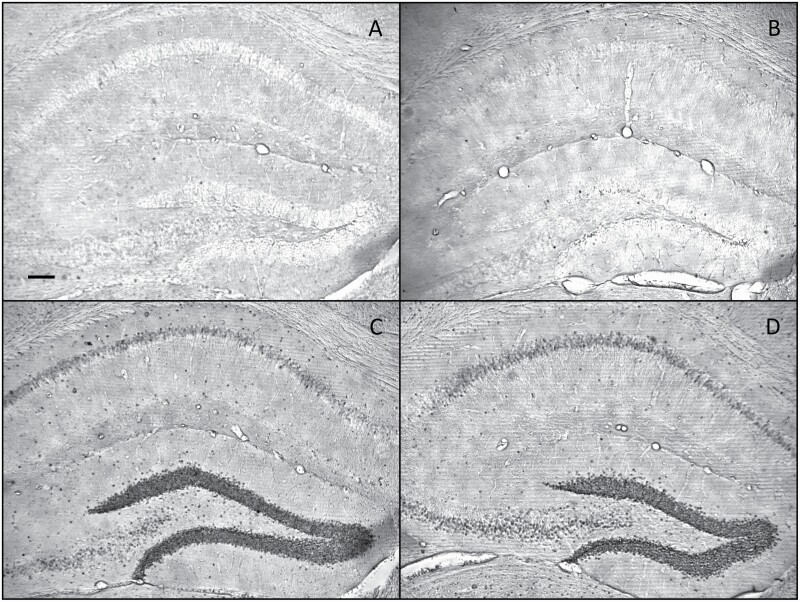
Cropped microscopy images showing c-fos induction in the hippocampus by treatment with saline (A), D-cycloserine (B), rapastinel (Glyx-13) + MK-801 (C), and D-cycloserine + MK-801 (D). Scale bar = 100 μm.

The c-fos brain mapping revealed a clear difference between the activation patterns of the different glutamatergic agents used. Rapastinel induced a significant c-fos expression in the prelimbic but not infralimbic area of the medial prefrontal cortex (Figure 1A–B). In accordance with previous studies (Gass et al., 1993), MK-801 induced widespread c-fos expression in numerous brain regions, including different subregions of the hippocampus (Figure 2A–C), cingulate (Figure 2E), and retrosplenial cortex (Figures 2F and 4). Animals receiving only Ro25-6981 exhibited no change in c-fos activity in the brain areas analyzed (Figure 2), as previously reported (Inta et al., 2009). Surprisingly, co-administration of rapastinel significantly enhanced the c-fos expression triggered by MK-801 in all examined regions investigated, except for the nucleus accumbens (Figure 2). In contrast, co-treatment of rapastinel with Ro25-6981 did not induce any measurable c-fos activation (Figure 2). D-Cycloserine induced a similar expression pattern in combination with MK-801 as rapastinel (Figures 3 and 5).

## Discussion

Rapastinel alone did not induce a significant c-fos expression beyond the prelimbic cortex, as previously reported (Liu et al., 2017). However, instead of diminishing, as expected, it strongly increased c-fos activation induced by the global antagonist MK-801. Although acting on different binding sites of the NMDAR, rapastinel and D-cycloserine exhibit similar effects on c-fos induction.

Our results illustrate an unexpected potential of rapastinel and D-cycloserine to activate, possibly by disinhibition, large cortical networks when added to MK-801. While such allosteric modulators alone disinhibit pyramidal neurons in select brain regions (Widman and McMahon, 2018), this effect on NMDARs appears to be dependent on dose and glutamate concentration (Donello et al., 2019). The currently reported enhanced network activity may be triggered by excessive glutamate release mediated by MK-801, which further increases NMDAR function induced by the examined allosteric modulators. We previously described a similar augmentation of MK-801 by GluN2-specific blockade: both GluN2B antagonism combined with MK-801 or MK-801 given to GluN2A KO mice stimulated specific forebrain areas, resulting in the “de novo” activation of specific brain regions (Inta et al., 2012). In that case, one possible explanation is that MK-801 acts not only on forebrain NMDARs containing the GluN2A/GluN2B subunits but mainly on predominantly extracortical GluN2C/GluN2D-containing NMDARs (Kotermanski, Wood and Johnson, 2009), suggesting a cumulative effect. However, more complex interactions, resulting from differential effects on NMDAR in different neuronal populations, may drive the excitatory-inhibitory balance in neuronal circuits, leading to the different c-fos activation patterns reported here. Whether this heightened signaling induced by allosteric modulators of NMDARs is a homeostatic reaction to the psychotomimetic effects of MK-801 currently remains unclear and will require further research.

Present results may have implications for understanding the role of glutamatergic agents in treating psychiatric disorders. Previous data indicated a role of the GluN2B subunit in mediating the antidepressant effect of both rapastinel and ketamine (Lang et al., 2018). We found that rapastinel, but not Ro25-6981, significantly increased c-fos induction in the prelimbic cortex. However, their combination did not result in an additional enhancement of c-fos expression, suggesting no cumulative effect between the 2 compounds. On the other hand, rapastinel was shown to alleviate schizophrenia-like abnormalities induced by MK-801 (Zhou et al., 2018). It is yet unclear if and how these behavioral effects relate to enhanced activation of psychosis-relevant brain regions, such as the retrosplenial cortex, observed after co-administration of both substances.

Our data identify for the first time, to our knowledge, complex interactions between different NMDAR modulators at the network level. Further functional studies may unravel the mechanisms underlying these effects and the potential therapeutic benefit or disadvantage of their combination.
